# Deformed wing virus can be transmitted during natural mating in honey bees and infect the queens

**DOI:** 10.1038/srep33065

**Published:** 2016-09-09

**Authors:** Esmaeil Amiri, Marina D. Meixner, Per Kryger

**Affiliations:** 1 Department of Agroecology, Aarhus University, Slagelse, 4200, Denmark; 2 Department of Biology, University of North Carolina, Greensboro, NC, 27403, USA; 3 LLH Bieneninstitut Kirchhain, Kirchhain, 35274, Germany

## Abstract

Deformed wing virus is an important contributor to honey bee colony losses. Frequently queen failure is reported as a cause for colony loss. Here we examine whether sexual transmission during multiple matings of queens is a possible way of virus infection in queens. In an environment with high prevalence of deformed wing virus, queens (n = 30) were trapped upon their return from natural mating flights. The last drone’s endophallus (n = 29), if present, was removed from the mated queens for deformed wing virus quantification, leading to the detection of high-level infection in 3 endophalli. After oviposition, viral quantification revealed that seven of the 30 queens had high-level deformed wing virus infections, in all tissues, including the semen stored in the spermathecae. Two groups of either unmated queens (n = 8) with induced egg laying, or queens (n = 12) mated in isolation with drones showing comparatively low deformed wing virus infections served as control. None of the control queens exhibited high-level viral infections. Our results demonstrate that deformed wing virus infected drones are competitive to mate and able to transmit the virus along with semen, which occasionally leads to queen infections. Virus transmission to queens during mating may be common and can contribute noticeably to queen failure.

Within the Hymenoptera, polyandry is uncommon and only known from the eusocial genera *Apis*, *Vespula*, *Pogonomyrmex* and *Atta*^1^. Extreme polyandry is omnipresent in the genus *Apis* (honey bees), where it has been well characterised^2^,^3^,^4^. In the honey bee, *Apis mellifera* L., mating occurs at a considerable distance to the queen’s colony, at so-called drone congregation areas (DCA)^5^. Very early in their life, young queens visit DCAs to mate in free flight with numerous drones, often taking several mating flights^2^,^6^.

Despites its benefits, multiple mating also introduces risks to honey bee queens, and consequently, to the colony. The queens face dangers from this mating behaviour, for instance through predation or adverse weather conditions^7^. Furthermore, given a certain proportion of infectious drones present in the DCA^8^, the risk of sexual transmission of disease increases with each additional mating^9^,^10^,^11^. Because honey bee drones die during copulation^5^, the evolution of sexually transmitted diseases in the genus *Apis* may be considered implausible. Nonetheless, a considerable number of studies found the reproductive organs of queens and drones to be frequently and highly infected with deformed wing virus (DWV), occasionally along with other viruses^8^,^12^,^13^,^14^,^15^,^16^,^17^,^18^.

Infection with DWV is common in honey bees^19^. It is known to be transmitted horizontally between adult bees via the fecal-oral pathway, and vertically between generations via egg or sperm^19^,^20^,^21^,^22^. In the absence of *Varroa destructor* mites, its prevalence and virus titres are usually low^19^,^23^,^24^ so that overt disease is rarely observed. In recent decades, the prevalence and virulence of DWV increased dramatically due to a change in its transmission route, to now include vector-mediated transmission^25^. The main trigger for this change was the host shift of this parasitic *Varroa* mite from the Asian *A. cerana* to *A. mellifera* and its subsequent worldwide spread^26^,^27^. As these mites depend on honey bee brood for their reproduction, infected mites can transfer large numbers of virus particles directly into the hemolymph of developing honey bee pupae^19^,^28^,^29^. It has also been shown that mite-mediated transmission of DWV considerably reduces viral diversity and selects for more virulent virus strains^23^,^25^,^26^,^30^. In addition, the *Varroa* mite may act as an intermediate host for DWV, although this role is not fully understood, yet^28^,^31^,^32^. Consequently, we today consider horizontal transmission by the mite vector the major reason for the high prevalence of DWV^19^,^22^,^33^.

*Varroa* mites are able to reproduce on both worker and drone brood on their new host *A. mellifera*, although with a strong preference for drone brood, in contrast to the mites’ exclusive reproduction on drone brood of the original host *A. cerana*^34^,^35^. As a result, drones originating from colonies with high mite infestation can be expected to show high levels of DWV infection^12^,^16^. In comparison, high levels of virus infection in queens cannot directly be explained by *Varroa* parasitisation, since the development time of the queen pupa is too short for the mite to complete its reproductive cycle. Therefore, one of the possible explanations for the reported high prevalence of DWV in queens could be virus transmission from infected drones via natural mating.

While it has previously been shown that infected drones are able to fly and frequently can reach DCAs^8^, there is little evidence that they successfully compete for mating with uninfected drones. Furthermore, it is not known if natural mating with an infected drone will result in transmission of pathogens to the queen causing infection, which then may be followed by subsequent vertical transmission of pathogens from parents to offspring^20^,^21^.

The presence and integrity of a queen is essential for a honey bee colony; therefore, queen health has a direct impact on colony health. Failure or loss of the queen is known as a discernible factor of colony loss^36^,^37^,^38^,^39^, and can happen due to several biotic and abiotic stressors^17^,^40^. In this paper, we investigate the role of mating in the transmission of DWV and present data to prove that (1) DWV infected drones are able to mate with queens, (2) DWV can be transmitted from drones to queens via natural mating, (3) within weeks after mating, DWV can be detected throughout the body of the queen.

## Results

### DWV in control queens

**Figure 1:**
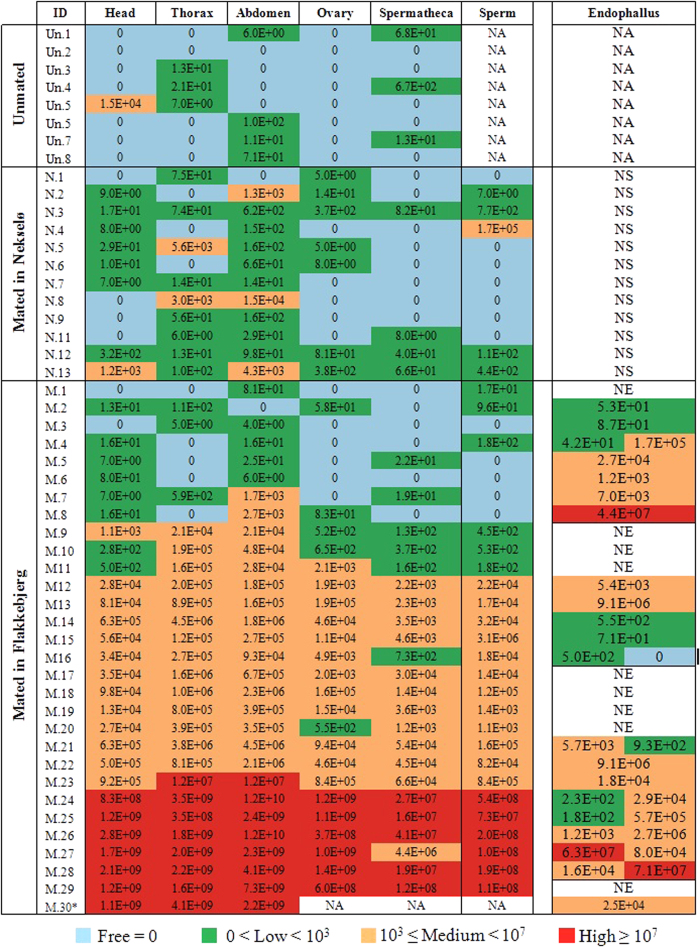
DWV titres in the head, thorax, abdomen, ovaries and spermatheca of each queen as along with the viral titres found in sperm taken from each mated queen. Furthermore, for each queen returning mated once or twice, the virus titres in the mating signs are recorded. * This queen was found dead and dissection of the internal organs was impossible. NS: Not Sampled, NA: Not applicable, NE: No endophallus upon return to hive.

All experimental queens were raised in the same nurse colony, and queen production was monitored closely for the presence of DWV and other viruses. All queen larvae and royal jelly samples collected from uncapped queen cells were free of DWV (data not shown). The tissues of most of the newly emerged queens either had no virus at all or contained low titres of DWV (for definitions of virus titres refer to Fig. 1). The titre of DWV in two of the newly emerged queens reached a medium level in the thorax and abdomen. In consequence, the nurse colony used to raise all the experimental queens had a low level of DWV^41^.

### DWV in drones and endophalli

All drones sampled from the healthy drone provider colonies on Nekselø were free of DWV. In contrast, the endophalli from nine drones captured in front of the high *Varroa* infested hives in Flakkebjerg (n = 47 drones) showed high levels of DWV.

Among the 30 queens allowed to mate in Flakkebjerg, nine returned from mating flights of more than 7 min without exhibiting mating signs upon their return. However, they started oviposition of fertilised eggs shortly after, thus confirming that they had successfully mated. From the 21 remaining queens, 29 mating signs were collected, either one or two endophalli from each queen (Fig. 1). The DWV levels in these endophalli were not significantly different from the virus levels in the endophalli collected from captured drones in front of the hives in Flakkebjerg (P = 0.897, ANOVA, df = 74).

### DWV in experimental queens

Among the unmated queens, virus levels were generally low and at a maximum reached a medium viral level. One of the unmated queens (Un.2, Fig. 1) was completely free of DWV in all tissue samples. The remaining seven queens of this group (Un.1, Un.3 to Un.8, Fig. 1) showed low titres only in the thorax (n = 3), abdomen (n = 4) and spermatheca (n = 1). A medium level of DWV infection was found in the head of a single queen (Un.5, see Fig. 1 for details).

The queens that mated with healthy drones on Nekselø, similarly only reached medium viral levels. Among the 72 analysed tissue samples from 12 queens mated on Nekselø (N.1 to N.12), only seven tissue samples were found to be infected at a medium level in the abdomen (n = 3), thorax (n = 2), head (n = 1) and sperm (n = 1) (Fig. 1). The remaining 65 tissue samples exhibited low virus levels or showed no DWV at all. Virus levels in the head, thorax and spermatheca were not significantly different between the Nekselø group and the unmated queens (P = 0.68, 0.445 and 0.57 respectively; ANOVA, df = 18). However, virus levels in the abdomen and ovaries of the mated Nekselø queens were significantly higher than those of the unmated queens (P = 0.0063 and 0.046 respectively; ANOVA, df = 18).

In contrast, we only observed high infection levels in the queens mated in Flakkebjerg, in seven of the 30 mated queens (M.24 to M.30, Fig. 1), with DWV occurring in all examined tissue types. Based on the pattern of infection, the Flakkebjerg group of queens was divided into three subgroups:

- This subgroup consisted of eight queens with little to no DWV titre in all examined tissues (M.1 to M.8, Fig. 1). Of these eight queens, six returned with a mating sign once, and one twice (Fig. 1). Three of these eight endophalli contained low virus levels, four expressed a moderate level, and one a high virus level. The sperm sampled from five queens were free of DWV, and the remaining three sperm samples had a low DWV titre. Only two of these eight queens expressed a medium virus level in the abdomen (see Fig. 1 for details).
- Within this subgroup fifteen queens expressed medium levels of DWV infection in the examined tissues (M.9 to M.23, Fig. 1), one of which (M.23) showed a high DWV titre only in the thorax and abdomen. Among these queens, five had a low viral load in the head, ovary, spermatheca and sperm (M.9, M.10, M.11, M.16, M.20), but their remaining tissues were infected with medium level virus titres. We had collected ten endophalli from these queens, one of which was free of virus, four expressed low viral titres and the other five showed a medium level of infection.
- The third subgroup was made up of the remaining seven Flakkebjerg queens (M.24 to M.30) and showed a high DWV load in most examined tissues. One of these queens was found dead (M.30) soon after the onset of oviposition, and a post-mortem RT-qPCR confirmed high viral titre of DWV in the head, thorax and abdomen. Of the eleven endophalli collected from six of these queens, two had low titres, seven a medium level, and two showed high viral titres (see Fig. 1 for details).

## Discussion

The objective of our experiment was to answer three key questions in regard to virus transmission as a potential cost in the polyandrous mating system of honey bee queens. Firstly, we investigated whether DWV infected drones are able to naturally mate with queens. Secondly, we examined if transmission of DWV occurs during natural mating. Finally, we detected DWV throughout the bodies of the mated queens shortly after mating.

It has been shown that *Varroa* mites favour drone brood for their reproduction^34^,^35^, therefore we expected a high virus prevalence in drone provider colonies in Flakkebjerg. Previous studies have shown that both newly emerged and mature drones can be infected with high titres of DWV especially in testes, mucus glands, seminal vesicles and sperm^12^,^15^,^16^. Our results confirmed that the Flakkebjerg drones were indeed infectious by having 9 out of 47 collected endophalli expressing high virus titres (C ≥ 10^7^).

Although adverse effects of *Varroa* parasitation on drone health and performance are well documented^35^,^42^,^43^, it is disputed if and how DWV infections impact drone fitness. In particular, the question of whether infected drones are capable of copulation is still under discussion^8^,^20^. Our data provide clear evidence, with high level of DWV infection found in three of the 29 endophalli collected from queens on return from mating flights. The data show that both infected and uninfected drones were present in the DCA visited by the 30 queens mated at Flakkebjerg, therefore we can conclude that infected drones are able to compete successfully for mating with healthy ones.

It is known that DWV can be transmitted during artificial insemination^20^,^21^, but proof was lacking that natural mating can also transmit the virus. In our experiment with 30 queens, we found high viral titres in five spermathecae and six sperm samples collected from queens’ spermathecae, providing evidence that this virus can be transmitted through natural mating. The ratio of infection (5 and 6, respectively, of 30) observed in our queens is slightly higher than that in the collected mating signs (3 of 29). Yet, we expected the majority of queens to copulate with at least one infected drone among the often more than 20 mating partners per queen^4^,^6^. We suggest that virus transmission during mating may often be ineffective, either because the drone’s fertility may be impaired by the infection, or because infection of a queen via sexual transmission is difficult *per se*. It may also be possible that some queens are not susceptible to DWV infection. In fact, one queen (M.8, Fig. 1), from which we collected an endophallus with a high viral titre, had no DWV in either the spermatheca or the sperm within and only showed a medium viral titre in the abdomen. Similar phenomena were reported previously about one of the queens artificially inseminated with DWV contaminated sperm^20^. We are unable to determine if this particular virus-infected drone in our experiment did not deposit any semen, or if the queen had a defence mechanism against DWV infection. It has been reported that drones whose endophalli were collected from a queen had no offspring^4^, suggesting a lack of sperm transfer.

Finally, we compared the virus infections of unmated queens with those of queens mated in Nekselø (healthy drones) and queens mated in Flakkebjerg (DWV infected drones). High titres of DWV only occurred in some queens of the Flakkebjerg group. Alternative transmission routes that could have led to this finding can be ruled out, since the two control groups (unmated queens and Nekselø queens) were maintained in the same apiary and mostly showed low infection levels. While horizontal transmission routes for DWV have been described^22^,^31^,^44^, it was recently shown that mechanisms to protect queens from infection may exist^45^. In the group of queens mating with healthy drones (Nekselø), the abdomen and thorax were more frequently infected than sperm and spermatheca, pointing towards horizontal transmission from worker contacts, rather than from drones during mating. The onset of oviposition is connected with an increased demand for feeding of the queen with royal jelly, supplied by workers^46^,^47^.

Discolouration of the ovaries, previously described as a pathological reaction to virus infection^17^, was not seen in our experiment. This was possibly due to terminating the experiment after only six weeks post queen mating, which might not be sufficient time for pathological ovary discoloration to become evident. One queen (M.30, Fig. 1) was found dead in her mating nuc soon after the onset of oviposition. While a full dissection and inspection of the ovaries from this queen could not be performed, high virus titres were measured in the head, thorax and abdomen (ovaries, spermatheca and sperm could not be analysed separately).

A healthy queen is an essential requirement for a strong honey bee colony. In the social context of the colony the queen seems to enjoy the benefit of a certain level of protection against infections. Experimental data suggest that queens and infected workers may avoid contact and only engage in trophallaxis when there is no alternative^45^. However, infection of queens may arise during sexual contacts with drones, and given the extreme degree of polyandry found in queens, seems to be an obvious route for DWV transmission, both between colonies and across generations^4^,^6^,^8^,^11^,^20^. High DWV titres from collected endophalli suggest a link between *Varroa* infestation of drone brood and virus transmission during mating. Consequently, infected queens might represent a potential source for distribution of DWV across honey bee populations worldwide^48^.

It is well documented that a DWV infection shortens the life expectancy of worker honey bees^33^, but currently no data are available on queen survival and queen performance in relation to an infection with DWV. Between 16% to 25% of lost colonies are directly attributed to queen events^37^,^38^,^39^,^49^,^50^. On the basis of our experimental data, we can develop the hypothesis that high titres of DWV following mating may contribute to explaining part of the queen failures in lost colonies.

## Methods

### Experimental design

In July 2012, a large group of sister queens were produced from a healthy colony following the standard procedure^51^, briefly: young larvae (12–24 hours old) were grafted into artificial queen cups and placed in a queenless nurse colony. Prior to grafting, the donor colony had been confirmed to be essentially free of *Varroa* mites (0 mites were found in a sample of 300 bees) and essentially free (no virus detected in a sample of 60 worker bees) of the most common bee viruses including: DWV, Black Queen Cell Virus (BQCV), Sacbrood Virus (SBV), Chronic Bee Paralysis Virus (CBPV) and ABPV complex (Acute Bee Paralysis Virus (ABPV), Kashmir Bee Virus (KBV) and Israeli Acute Paralysis Virus (IAPV)) using the technique previously described^41^.

After six days, fifteen uncapped queen cells containing larvae and royal jelly were sampled and stored at −80 °C. The rest of the queen cells were caged individually (post capping) and transferred to an incubator. Upon emergence, 10 queens were also sampled and stored at −80 °C. The remaining 50 queens were introduced into mating nucs containing 200–250 worker bees^51^. Following the queen introduction, the mating nucs were divided into three groups.

Mating nucs of group 1 (n = 8) were placed in the experimental apiary Flakkebjerg. Queens in this group were treated twice with CO_2_ to stimulate oviposition without mating^52^. Each queen was returned to her own mating nuc following CO_2_ treatment. The entrance of each mating nuc was covered with a queen excluder that allowed workers to pass, but prevented the queens from exiting.

Mating nucs of group 2 (n = 12) were placed on the island mating station, Nekselø, and the queens were allowed to mate freely. For the purpose of mating, an adequate number of mature drones were provided by twelve colonies that are regularly inspected for health and treated against *Varroa* mites. The viruses’ status test was performed by sampling 10 to 15 mature drones upon their return to each drone provider hive. The drones from each hive were pooled, freeze-dried and homogenised, and analysed for viruses^41^, confirming that they had no viruses or only low titres of DWV and ABPV complex. In order to have the same foraging environment following the queens ovipositing, all mating nucs were transferred from Nekselø to Flakkebjerg and maintained together with groups 1 and 3.

Mating nucs of group 3 (n = 30) were also placed in Flakkebjerg and equipped with queen excluders in front of the mating nucs, as explained above (Mating nucs group 1). These mating nucs were continuously observed on sunny afternoons, between 13:00 and 18:00. When a queen appeared at the entrance for an orientation or a mating flight, the queen excluder was removed and as soon as she departed, the excluder was reinstalled to prevent her unobserved re-entry^47^. Upon returning, each queen was captured if carrying a mating sign, which consisted of her last mating partner’s endophallus. All mating signs were carefully removed in the field using sterile forceps, stored individually in Eppendorf tubes on ice, and transferred to −80 °C at the end of the day. Following the removal of the mating sign, each queen was released into her own mating nuc. Every queen was permitted to fly as often as she wanted and the duration of each flight was recorded, in order to assure that a flight of a duration sufficient for mating had occurred (>7 minutes).

The drone provider colonies in Flakkebjerg (5 colonies) had not been treated against *Varroa* mites for the last three years before the experiment, and were allowed to produce drones freely. Prior to the experiment, analysis of fifty worker bees per drone colony confirmed that they all had high levels of DWV, but only showed a minor infection with ABPV complex viruses. In addition, mature drones (n = 47) were captured upon returning to the hives, and their endophalli were individually analysed for viruses.

All the queens from the three experimental groups were removed from their mating nucs six weeks after the onset of oviposition. The queens were anaesthetised using carbon dioxide, sacrificed and dissected^53^ to generate tissue samples of the head, thorax, abdomen, ovaries, spermatheca and sperm. Sperm was extracted from the spermatheca with a hair capillary. All tissue samples were stored at −80 °C.

### Molecular Assays

All tissues except the spermatheca and sperm were freeze-dried for three days at 0.0009hPa and −93 °C. Lyophilised tissue samples were crushed and homogenised for 30 s at 500 Hz. Micro-pestles were used to homogenise the spermatheca and sperm tissues. Total RNA was extracted from each sample using NucleoMag 96 RNA Kit. A two-step real time RT-qPCR assay was carried out to quantify the viral load of DWV, SBV, and the ABPV complex as described previously^14^. The quality of the extracted RNA and the efficiency of the PCR reactions in experimental samples were confirmed by amplification of the reference gene β-Actin. Viruses of the ABPV complex were not detected in any tissue samples from queens or drones. SBV was only detected in a few (n = 5) queen abdomen tissues.

### Data analysis

Virus copy numbers in each sample were quantified using the absolute quantification method described previously^14^. One-way analysis of variance (ANOVA) was used for analysis of normalised data for each tissue sample among the three experimental groups. Multiple comparisons were performed with Tukey’s test. Data organisation was performed using Excel and R version 3.1.3.

## Additional Information

**How to cite this article**: Amiri, E. *et al.* Deformed wing virus can be transmitted during natural mating in honey bees and infect the queens. *Sci. Rep.* **6**, 33065; doi: 10.1038/srep33065 (2016).
